# Atomic-Layer-Deposition Assisted Formation of Wafer-Scale Double-Layer Metal Nanoparticles with Tunable Nanogap for Surface-Enhanced Raman Scattering

**DOI:** 10.1038/s41598-017-05533-4

**Published:** 2017-07-12

**Authors:** Yan-Qiang Cao, Kang Qin, Lin Zhu, Xu Qian, Xue-Jin Zhang, Di Wu, Ai-Dong Li

**Affiliations:** National Laboratory of Solid State Microstructures and Department of Materials Science and Engineering, College of Engineering and Applied Sciences, Collaborative Innovation Center of Advanced Microstructures, Nanjing University, Nanjing, 210093 People’s Republic of China

## Abstract

A simple high-throughput approach is presented in this work to fabricate the Au nanoparticles (NPs)/nanogap/Au NPs structure for surface enhanced Raman scattering (SERS). This plasmonic nanostructure can be prepared feasibly by the combination of rapid thermal annealing (RTA), atomic layer deposition (ALD) and chemical etching process. The nanogap size between Au NPs can be easily and precisely tuned to nanometer scale by adjusting the thickness of sacrificial ALD Al_2_O_3_ layer. Finite-difference time-domain (FDTD) simulation data indicate that most of enhanced field locates at Au NPs nanogap area. Moreover, Au NPs/nanogap/Au NPs structure with smaller gap exhibits the larger electromagnetic field. Experimental results agree well with FDTD simulation data, the plasmonic structure with smaller nanogap size has a stronger Raman intensity. There is highly strong plasmonic coupling in the Au nanogap, so that a great SERS effect is obtained when detecting methylene blue (MB) molecules with an enhancement factor (EF) over 10^7^. Furthermore, this plasmonic nanostructure can be designed on large area with high density and high intensity hot spots. This strategy of producing nanoscale metal gap on large area has significant implications for ultrasensitive Raman detection and practical SERS application.

## Introduction

Raman spectroscopy is one of the most promising molecule detection techniques because of its unique specificity for molecules and the vibration peaks of chemical bonds. However, in most cases, Raman scattering of organic molecules is too weak to be detected due to its extremely small scattering cross section^1^. Fortunately, it was found that plasmonic coupling between some metal (Au, Ag or Cu) can produce strong local electromagnetic field, called electromagnetic hot spots. If the detected molecules are adsorbed at these hot spots, the Raman intensity can be enhanced by tens of orders of magnitude^2–5^. This technology is called as surface enhanced Raman scattering (SERS), which has attracted increasing attention in diverse areas, including chemistry, biology, food safety and biomedical diagnostics, etc.^6–10^. However, fabricating perfect plasmonic nanostructures with high density and high intensity hot spots has been one of the major challenges in SERS research.

So far, numerous efforts have been made to design various forms of noble metallic nanostructures as SERS substrates, in which colloidal solution is widely employed to systhesis the metallic nanostructures, including nanoparticles, nanocubes and nanowires^3, 11, 12^. These nanostructures can show an extremely large enhancement factor and even a great potential in single molecule detection^6, 13^. However, it is very difficult to control the position and interval of adjacent metal nanoparticles with colloidal solution method. As a result, it’s hard to get a uniform and repeatable singal by these SERS substrates. At the same time, it is a challenge to achieve clean chemical background and wafer-scale fabracation with colloidal solution method. Mirco-nano fabrication technology, such as E-beam lithology (EBL), can be used to make diverse nanostuctures. In this way, many effective noble metal nanostuctures have been designed as SERS substrates^14–17^, such as M-shaped grating^16^ and hexagonal nanodisk array^17^. These stuctures can exhibit both strong and uniform Raman signal. Hoverver, using micro-nano fabrication technology usually needs a very complicated process and extremely high cost. In addition, the nanostructure can be only designed on a very small area. Consequently, this method can’t be applied for wafer-scale fabrication too. Therefore, it’s urgent and important for practical SERS application to develop a facile approach for wafer-scale SERS substrate fabrication.

Atomic layer deposition (ALD) is a novel thin film deposition technique based on sequential self-limited and complementary surface chemisorption reactions, which has attracted increasing attention in synthesis and surface engineering of complex nanostructures in recent years^18, 19^. ALD has shown great prospects in various applications, such as lithium-ion batteries^20^, supercapacitors^21, 22^, catalysis^23^, solar energy conversions^24^, and photonics^18^. ALD is a powerful and useful technology to fabricate the plasmonic metal nanogap for SERS application^25–27^. Moreover, ALD coatings can protect the SERS substrate against oxidation and contaminant, improving the stability of SERS substrate^28, 29^.

Herein, we present a very simple but effective approach to fabricate the wafer-scale SERS structure of Au NPs/nanogap/Au NPs. A sacrificial ultrathin Al_2_O_3_ isolation layer is introduced into the double-layer stacked Au NPs by ALD, forming Au NPs/Al_2_O_3_/Au NPs structure. Then, partial Al_2_O_3_ between Au NPs is removed by chemical etching, producing the nanogap between Au NPs. The nanogap size can be easily and precisely tuned to nanometer scale by adjusting the thickness of ALD Al_2_O_3_ layer. The Raman results show that Au NPs/nanogap/Au NPs structure has a great SERS effect, and the Au NPs/nanogap/Au NPs structure with smaller nanogap has a stronger Raman intensity. This approach with no high-end micro-nano fabrication technology is far simple but effective, which shows great potential for wafer-scale SERS substrate fabrication and practial SERS application.

## Results

### Fabrication process of Au NPs/nanogap/Au NPs

**Figure 1:**
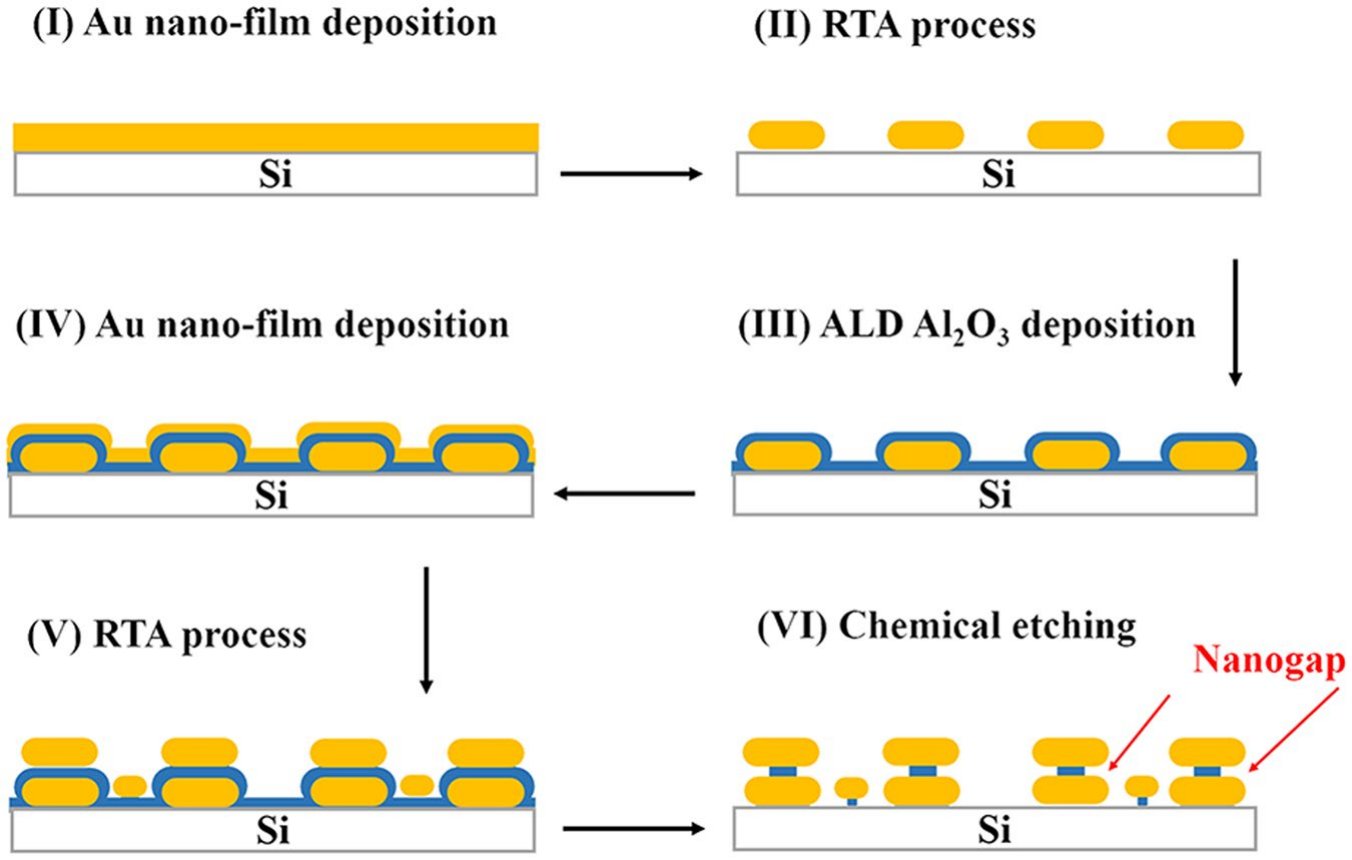
Schematic of fabrication process of Au NPs/nanogap/Au NPs structure. (**I**) Thin Au film is deposited on silicon substrate by sputtering; (**II**) Au NPs are formed by RTA process; (**III**) Ultrathin Al_2_O_3_ is coated on the surface of Au NPs via ALD process; (**IV**,**V**) Another thin Au film is deposited on the Al_2_O_3_ coated Au NPs, followed by RTA process to form a stacking structure of Au NPs/Al_2_O_3_/Au NPs; (**VI**) Partial Al_2_O_3_ is etched by 5% KOH solution, forming the Au NPs/nanogap/Au NPs structure.

Figure 1 shows the schematic of fabrication process of SERS substrate of Au NPs/nanogap/Au NPs. Firstly, a thin Au film is deposited on the silicon substrate using magnetron sputtering (Quorum, Q150TS) with current of 5 mA (step I). Au NPs arrays are formed by rapid thermal annealing (RTA) of Au nanofilm at 500 °C for 30 s (step II). Then, an ultrathin (2–20 nm) Al_2_O_3_ film is deposited via an ALD process at 200 °C, resulting in excellent surface coating on the Au NPs arrays (step III). Next, repeat the step I and II, the second thin Au nanofilm is deposited on the Al_2_O_3_ coated Au NPs arrays, and subsequent second RTA process is again carried out to form the second Au NPs arrays on the original Au NPs arrays isolated by Al_2_O_3_ layer (step IV and V). The sputtering time is optimized to be 60 s and 70 s for step I and IV, respectively, to achieve the best SERS effect, as shown in Fig. S1. Here, Au NPs/Al_2_O_3_/Au NPs composite nanostructure is gained. Finally, Au NPs/Al_2_O_3_/Au NPs structure is immersed in the 5% KOH solution for 120 s to etch partial sacrificial Al_2_O_3_, forming the Au NPs/nanogap/Au NPs structure (step VI). A well-formed nanogap-rich Au NPs stacking structure can include a high density of nanogaps for localization of electromagnetic field and thus highly sensitive hot spots for SERS.

In the above schematic, the nanogap size is determined by the thickness of sacrificial Al_2_O_3_ layer, which can be precisely and easily controlled by the number of ALD deposition cycles. Moreover, very uniform, dense and precisely controllable metal nanogap can be achieved due to the angstrom scale thickness resolution, uniformity, as well as nearly perfect conformality of ALD process^30^. Furthermore, all the technologies employed here can be performed over a very large wafer, which is very difficult to be accomplished by chemical self-assembly and usual lithography technology.

### Finite-difference time-domain simulation

**Figure 2:**
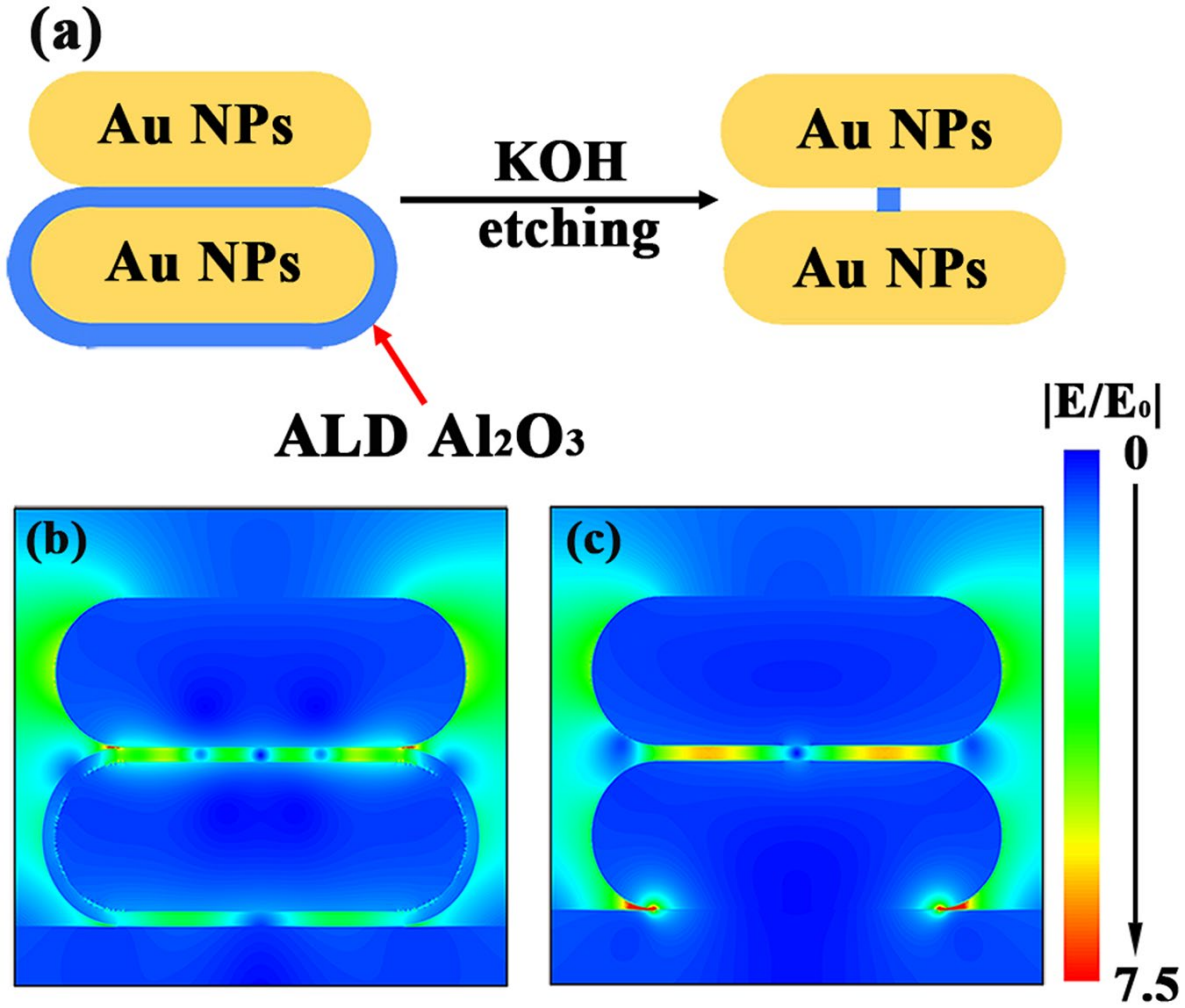
(**a**) FDTD simulation model of Au NPs/Al_2_O_3_/Au NPs and Au NPs/nanogap/Au NPs. FDTD simulated electric field distribution of (**b**) Au NPs/Al_2_O_3_(5 nm)/Au NPs and (**c**) Au NPs/nanogap(5 nm)/Au NPs. The excitation laser wavelength used in FDTD is 633 nm.

Theoretical simulation based on finite-difference time-domain (FDTD) method was performed to investigate the plasmonic coupling in Au NPs/Al_2_O_3_/Au NPs and Au NPs/nanogap/Au NPs nanostructures. The excitation laser wavelength used in FDTD simulation is 633 nm. As shown in Fig. 2a, FDTD simulation model consists of vertically stacked two Au NPs (flat sphere), the bottom one is coated by a uniform thin Al_2_O_3_. After KOH etching, only a little Al_2_O_3_ with a diameter of 10 nm is left in the centre to support the stacked Au NPs structure. Figure 2b and c show the simulated electric field distribution in Au NPs/Al_2_O_3_/Au NPs and Au NPs/nanogap/Au NPs, respectively. For Au NPs/Al_2_O_3_/Au NPs, except the field near the tip of Au NPs is a little enhanced caused by tip effect, most of the enhanced electric field locates at the Al_2_O_3_ layer region between Au NPs due to the plasmonic coupling of Au NPs. Although there is strong plasmonic coupling in Al_2_O_3_ layer between Au NPs, this region is useless because the existing Al_2_O_3_ would block the analytes from getting into this region. After KOH etching process, firstly, the gap area with strong electromagnetic field is exposed to the analytes. In addition, the electric field in Au NPs gap area is enhanced slightly compared to the sample before etching. Therefore, the chemical etching process is crucial to achieve a high SERS effect, exposing hot spots to analytes.

**Figure 3:**
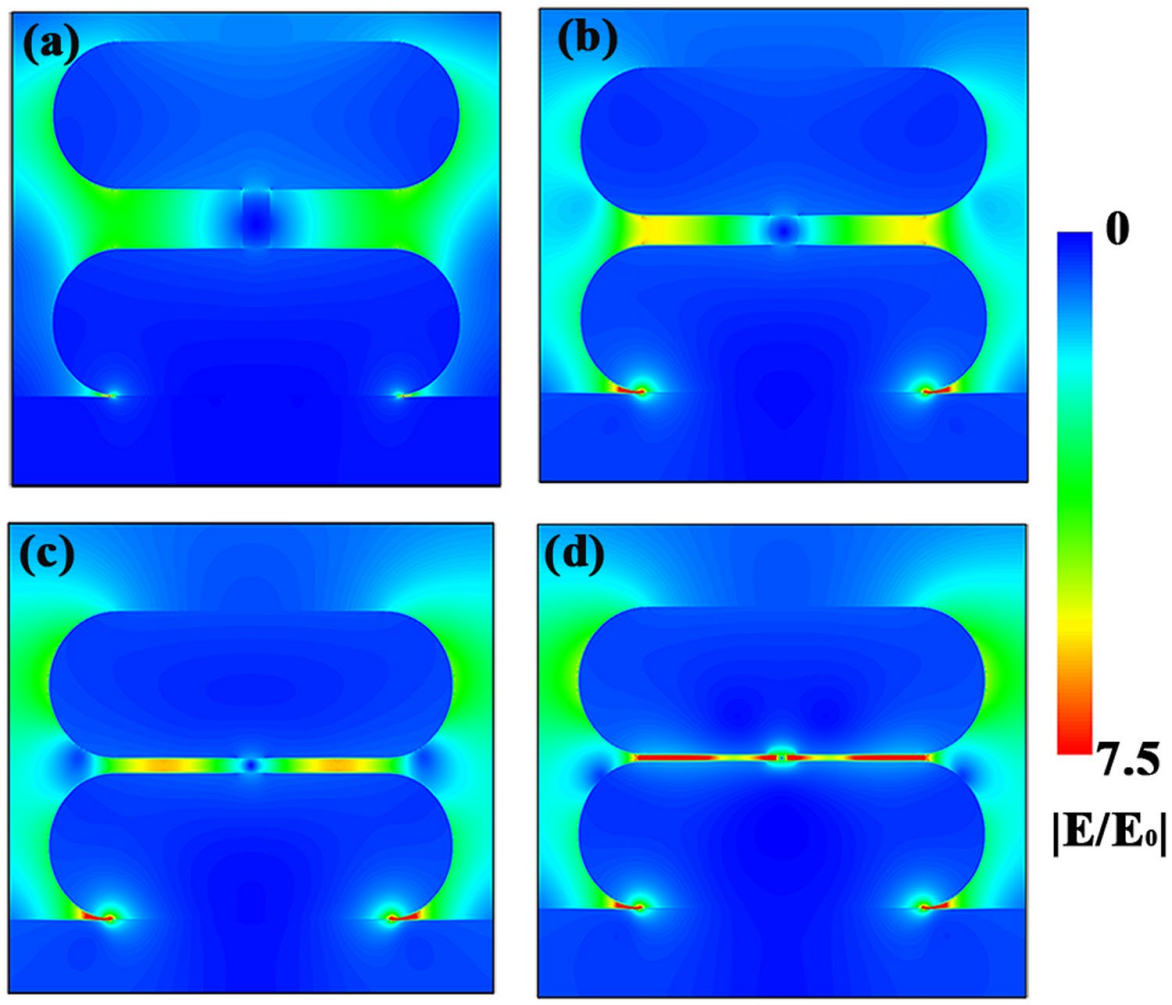
FDTD simulated electric field distribution of Au NPs/nanogap/Au NPs with various gap sizes: (**a**) 20, (**b**) 10, (**c**) 5, and (**d**) 2 nm.

Moreover, in order to investigate the effect of gap size on the enhancement of electric field, the electric field distribution of Au NPs/nanogap/Au NPs structures with various gap sizes from 2–20 nm was also simulated by FDTD method. Figure 3 shows the simulated electric field distribution for the Au NPs/nanogap/Au NPs with gap size of 20, 10, 5, and 2 nm, respectively. It can be easily seen that most of enhanced electromagnetic field locates at the gap area for all the samples, and the structure with smaller gap exhibits the larger electromagnetic field. Furthermore, the enhancement of the electromagnetic field is very limited when the gap size of Au NPs is very large (~20 nm), as shown in Fig. 3a. The comparison of field enhancement factor |E/E_0_| with various gap sizes can be found in Fig. S2. These results indicate that the plasmonic coupling depends strongly on the distance of Au NPs, where Au NPs/nanogap/Au NPs with 2 nm-nanogap exhibits the largest |E/E_0_| of 9.8.

Above results of FDTD simulation testify the feasibility of our strategy to build the Au NPs/nanogap/Au NPs structure for SERS. This strategy is based on the combination of ALD and chemical etching process. ALD is a powerful technology to perfectly coat nanostructures and precisely tune the distance of two layer Au NPs to nanometer scale, while chemical etching process could expose the strong electromagnetic field in gap area to the analytes.

### Microstructures observations

**Figure 4:**
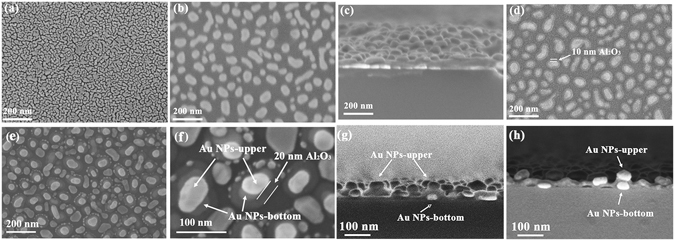
FE-SEM images of (**a**) as-deposited Au film, (**b**) surface and (**c**) cross-section of Au NPs, (**d**) 10 nm Al_2_O_3_ coated Au NPs, (**e**) Au NPs/Al_2_O_3_(20 nm)/Au NPs structure, (**f**) a high resolution image of Au NPs/Al_2_O_3_/Au NPs structure. FE-SEM cross-section images of (**g**) Au NPs/Al_2_O_3_/Au NPs structure and (**h**) Au NPs/nanogap/Au NPs structure.

Figure 4 shows the FE-SEM images of the SERS substrate during the fabrication process. Firstly, we can see that as-deposited thin Au nanofilm (60 s) is discontinuous (Fig. 4a). After annealing, Au thin film is transformed into Au NPs (Fig. 4b). From the cross-section SEM image, it can be found that the formed Au NPs is not sphere but flat sphere, as shown in Fig. 4c. ALD is known as a very powerful technology to coat the nanostructure uniformly. After ALD Al_2_O_3_ deposition process, a very uniform Al_2_O_3_ layer (~ 10 nm) is formed on Au NPs arrays (Fig. 4d). Figure 4e and f exhibit the SEM images of the substrate after the formation of second Au NPs layer, it can be found that there are two contrasts for Au NPs. The dark ones on the bottom are coated by uniform Al_2_O_3_ film, indicating the thickness of Al_2_O_3_ is around 20 nm. The Al_2_O_3_ coating is nonconductive, resulting in the dark contrast. We can easily discern that the light ones mostly locate on the top of the first Au NPs layer, indicating the formation of Au NPs/Al_2_O_3_/Au NPs structure.

It is more convenient to observe the Au NPs stack structure from the cross-section SEM image. For Au NPs/Al_2_O_3_/Au NPs, as shown in Fig. 4g, it can be easily seen there are two vertically stacked Au NPs layers, which are separated by a uniform Al_2_O_3_ layer. Figure 4h shows the cross-section image after KOH etching process, it can be found that most of Al_2_O_3_ is etched compared to the Au NPs/Al_2_O_3_/Au NPs structure. However, the stacked two Au NPs array is remained, indicating the formation of Au NPs/nanogap/Au NPs structure.

### SERS characterizations

**Figure 5:**
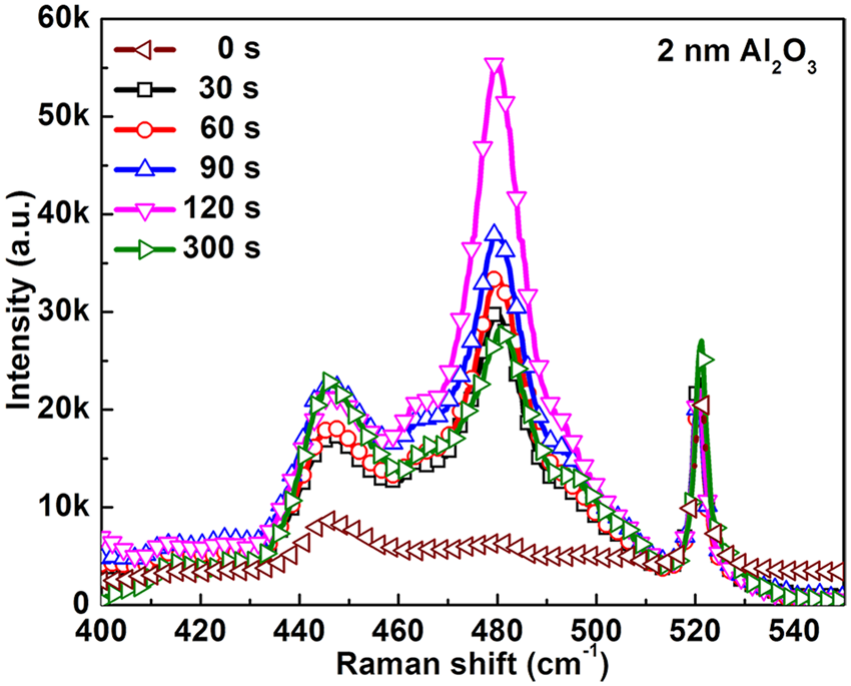
Raman spectra of MB molecules adsorbed on Au NPs/2 nm-Al_2_O_3_/Au NPs structure with various KOH etching time from 0 s–300 s.

Figure 5 shows the Raman spectra of MB molecules adsorbed on Au NPs/2 nm-Al_2_O_3_/Au NPs structure with various KOH etching time from 0 s–300 s. The most prominent features in the Raman spectra appear at 446 and 479 cm^−1^, assigned to the C-N-C skeletal bending of MB^31^. And the 520.7 cm^−1^ peak comes from Si substrate. It can be found that Raman signal of MB is very weak for the Au NPs/Al_2_O_3_/Au NPs structure without KOH etching. After only 30 s etching, forming the Au NPs/nanogap/Au NPs structure, the sample shows a much stronger Raman intensity. It can be ascribed to the fact that MB molecules can run into the Au NPs gap area after etching partial Al_2_O_3_. According to the simulation in Figs 2 and 3, Au NPs gap area shows much stronger electromagnetic field than other area. Moreover, the Raman signal becomes stronger and stronger with extending the etching time from 30 s to 120 s. Because more Au NPs gap area could be exposed with longer etching time. However, the Raman signal decreases dramatically for the 300 s etched sample, which may be caused by the damage of effective SERS structure.

**Figure 6:**
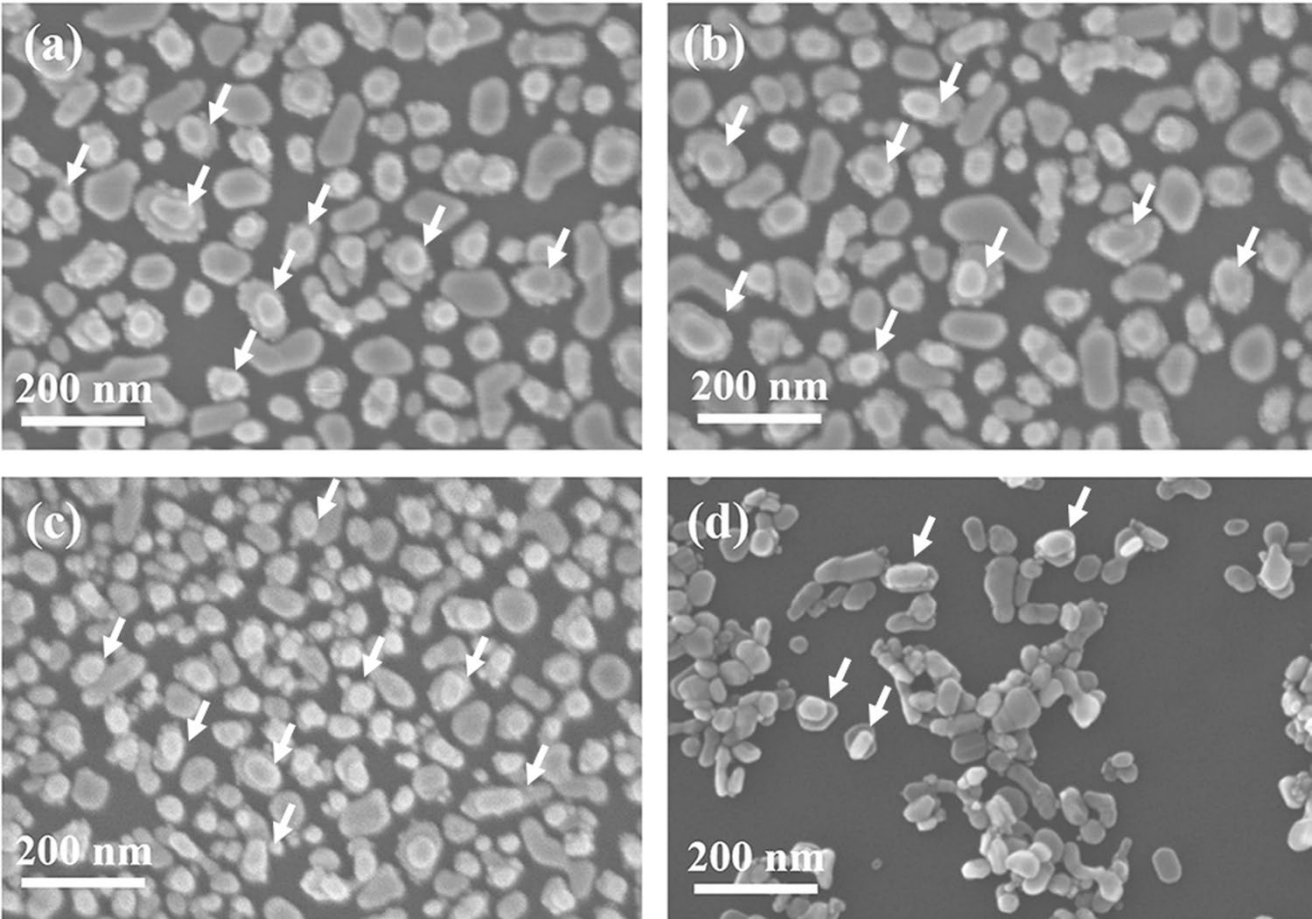
FE-SEM images of Au NPs/nanogap/Au NPs structure with (**a**) 30 s, (**b**) 90 s, (**c**) 120 s and (**d**) 300 s KOH etching.

Therefore, SEM was performed to observe the morphology change during the etching process, as shown in Fig. 6. At the beginning of etching (30–120 s), it can be seen that all samples exhibit the similar morphology. Stacked Au NPs structure can be easily observed, as marked by the arrows in Fig. 6a–c. In contrast, 300 s etched sample shows the extremely different morphology, as shown in Fig. 6d, most of the substrate surface is exposed and some Au NPs aggregate together. It can be ascribed to the fact that excessive KOH etching will consume the Al_2_O_3_ layer and also the SiO_2_ on the substrate, resulting in the Au NPs dropping off the substrate. As a result, the effective SERS structure would be destroyed. Although the SERS structure was damaged after excessive etching, the Raman intensity is still stronger than Au NPs/Al_2_O_3_/Au NPs. This is because that there are still some Au NPs/nanogap/Au NPs structures left on the substrate, as marked by the arrows in Fig. 6d. Therefore, it’s important to control the etching time, 120 s was chosen as etching time in the following experiment.

**Figure 7:**
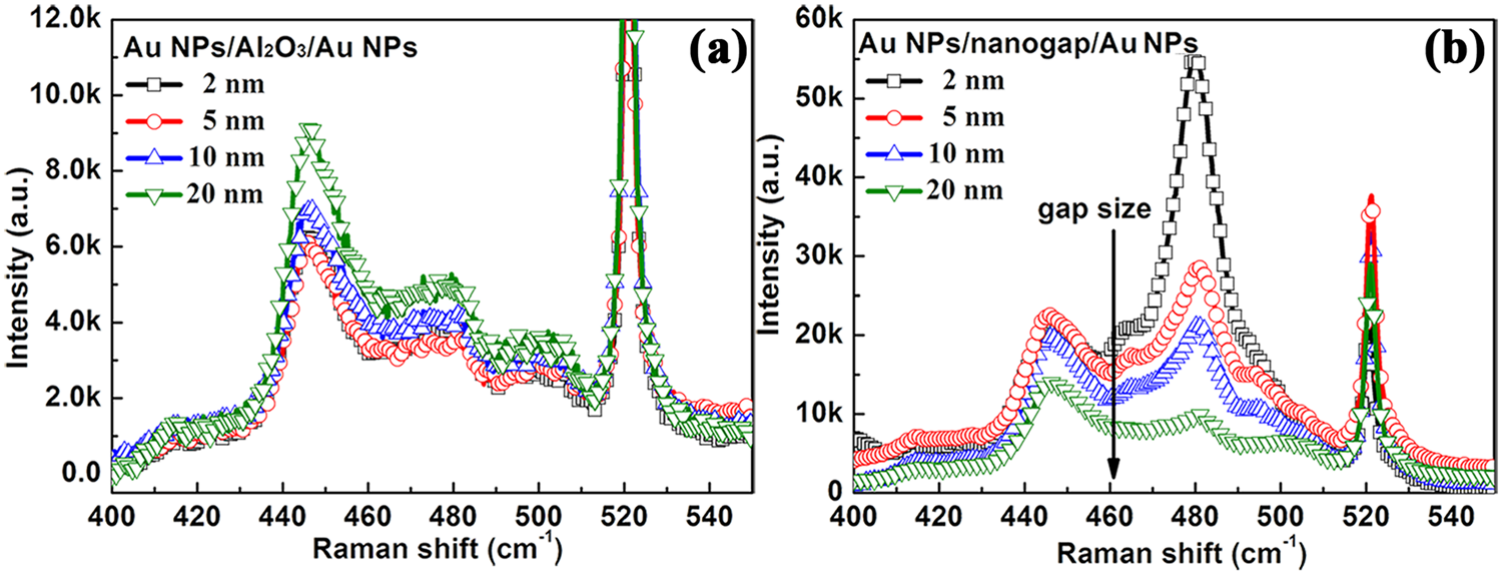
Raman spectra of MB molecules adsorbed on (**a**) Au NPs/Al_2_O_3_/Au NPs and (**b**) Au NPs/nanogap/Au NPs with various Al_2_O_3_ thickness.

In order to explore the effect of the gap size on the SERS effect, Raman spectra of MB molecules adsorbed on Au NPs/nanogap/Au NPs structure with different nanogap size were also performed. Firstly, Au NPs/Al_2_O_3_/Au NPs structures with different Al_2_O_3_ thickness were used as SERS substrates. All the Au NPs/Al_2_O_3_/Au NPs structures exhibit nearly the same Raman intensity, as shown in Fig. 7a, indicating that the thickness of Al_2_O_3_ has no influence on the SERS effect for Au NPs/Al_2_O_3_/Au NPs structure. In contrast, the Raman intensity increases dramatically with decreasing the nanogap size for Au NPs/nanogap/Au NPs structure, as shown in Fig. 7b. It can be ascribed to the fact that gap area with smaller gap size could exhibit the stronger electromagnetic field, as illustrated in FDTD simulation (Fig. 3). Fig. S3 compares the Raman intensity (479 cm^−1^) of Au NPs/Al_2_O_3_/Au NPs and Au NPs/nanogap/Au NPs, it can be easily seen that all the samples exhibit an improved Raman intensity after etching. Experimental Raman results agree well with FDTD simulation data (Figs 2 and 3).

**Figure 8:**
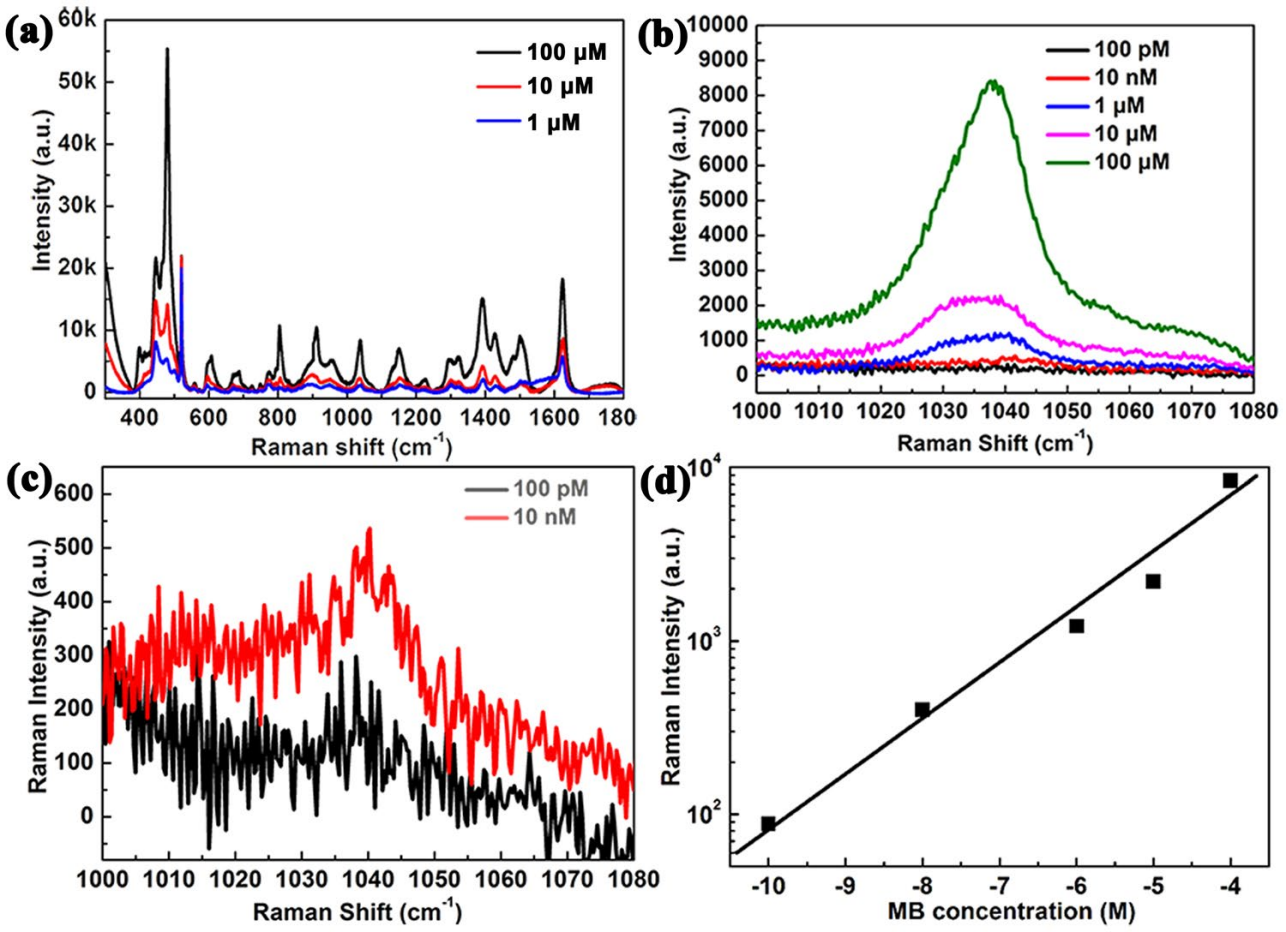
(**a**–**c**) Raman spectra of MB molecules adsorbed on Au NPs/nanogap (2 nm)/Au NPs structure with different MB concentration. (**d**) The dependence of Raman peak intensity at 1039 cm^−1^ on MB concentration.

In addition, this Au NPs/nanogap/Au NPs structure can be used in quantitative analysis of molecules. The SERS spectra of MB were collected from Au NPs/nanogap (2 nm)/Au NPs with different MB concentrations (from 10^−4^ M to 10^−10^ M), as showed in the Fig. 8a–c. It can be found that the intensity of SERS signature peak is reduced gradually with decreasing the concentration. The Raman signature of MB can be identified clearly. Even at an extremely low concentration of 10^−10^ M, a weak signature peak of MB at 1039 cm^−1^ still can be identified. Furthermore, the linear fitting between the Raman intensity of 1039 cm^−1^ and MB concentration was performed, as plotted in Fig. 8d. Above quantitative analysis results indicate that Au NPs/nanogap/Au NPs structure has strong detecting ability by SERS signature at nanomolar level.

In order to estimate the enhancement factor (EF) of Au NPs/nanogap (2 nm)/Au NPs, a clean Si substrate was employed as a reference. The EF of SERS can be calculated using the following equation:^32^

$${\rm{EF}}=({{\rm{I}}}_{{\rm{SERS}}}/{{\rm{I}}}_{{\rm{normal}}})\times ({{\rm{N}}}_{{\rm{normal}}}/{{\rm{N}}}_{{\rm{SERS}}})$$

where I_SERS_ and I_normal_ correspond to Raman intensity of 1039 cm^−1^ peak for 10^−10^ M MB on Au NPs/nanogap(2 nm)/Au NPs and 10^−3^ M MB on Si substrate, respectively. And N_normal_ and N_SERS_ are the number of MB molecules probed in Si substrate and Au NPs/nanogap (2 nm)/Au NPs, respectively. The EF of Au NPs/nanogap (2 nm)/Au NPs is evaluated to be 2.2 × 10^7^. In addition, The EF increases dramatically as probe molecule concentration decreases, as shown in Fig. S4.

Moreover, this strategy of combining ALD and chemical etching can be extended to other systems, such as metal NPs/nanogap/metal nanowires, metal NPs/nanogap/metal film, and nanostructured metal film/nanogap/metal film. Fig. S5 and S6 show the SEM image and Raman spectra of Au NPs/nanogap/Au film, respectively. Raman intensity of MB is significantly enhanced after KOH etching for Au NPs/Al_2_O_3_/Au film, similar to the Au NPs/Al_2_O_3_/Au NPs.

## Conclusions

In summary, we have successfully presented a simple high-throughput approach to fabricate wafer scale Au NPs/nanogap/Au NPs structure for SERS. This SERS substrate can be prepared feasibly by the combination of rapid thermal annealing, ALD and chemical etching process. The nanogap size can be easily and precisely controlled to nanometer scale by adjusting the thickness of sacrificial ALD Al_2_O_3_ layer. Chemical etching process could expose the strong electromagnetic field area to analytes. The plasmonic structure with smaller nanogap size shows better SERS effect, where Au NPs/nanogap/Au NPs structure with 2 nm gap size shows the best SERS effect to detect the MB molecules with EF over 10^7^. Furthermore, experimental Raman data agree well with FDTD simulation results, Au NPs/nanogap/Au NPs structure with smaller gap exhibits the larger electromagnetic field. This method of precisely producing nanoscale metal gap on large area has significant implications for ultrasensitive Raman detection and practical SERS application.

## Methods

### Chemicals and Materials

In ALD process, trimethylaluminium (TMA) (6 N, Nata Opto-electronic Material) and deionized water were used as Al precursor and oxygen source, respectively. High Purity N_2_ (5 N) was used as carrier and purge gas. The silicon (p-type, 100) was used as substrate. Organic dye of methylene blue (MB, C_16_H_18_ClN_3_S) was utilized for Raman detection. All chemicals were used without further purification.

### ALD deposition parameter of Al_2_O_3_

Al_2_O_3_ ultrathin films were deposited on Au NPs at 200 °C in a commercial Picosun SUNALE™ R-200 ALD reactor. TMA and deionized water were used as precursors, respectively. Both sources were kept at room temperature. Pulse time of both precursors was 0.1 s, followed by a 4 s N_2_ purge step to blow away redundant precursors and byproducts. The typical growth rate of Al_2_O_3_ is ~0.1 nm per cycle.

### Microstructure characterization

Field emission scanning electron microscopy (FE-SEM, Ultra55, ZEISS) was performed to observe the microstructure and morphology of the substrate after each step.

### SERS measurements

The solution of MB with 10^−4^ M was prepared, and this solution can be diluted to different concentration (10^−5^–10^−10^ M) for a quantitative analysis of SERS. Differently prepared substrates were immersed into the MB solution for 30 min. Self-assembled monolayer of MB can be adsorbed on the substrates after final water wash process. Raman spectra of MB were collected by a confocal Raman microscope (Horiba Jobin Yvon HR800 spectrometer) with excitation laser wavelength of 632.8 nm. An objective lens is employed to focus the excitation laser on the substrate and collect the Raman signal.

## Electronic supplementary material

Supplementary Information
