# Supplementary material for: Atomic-Layer-Deposition Assisted Formation of Wafer-Scale Double-Layer Metal Nanoparticles with Tunable Nanogap for Surface-Enhanced Raman Scattering
Source: Sci Rep. 2017 Jul 12;7:5161. doi: 10.1038/s41598-017-05533-4 (PMC5507941; doi:10.1038/s41598-017-05533-4)
Supplement: Supplementary file 1 — Supplementary Information [file 41598_2017_5533_MOESM1_ESM.pdf]

Supplementary information

Atomic-Layer-Deposition Assisted Formation of Wafer-Scale Double-Layer Metal Nanoparticles with Tunable Nanogap for Surface-Enhanced Raman Scattering

Yan-Qiang Cao*, Kang Qin, Lin Zhu, Xu Qian, Xue-Jin Zhang, Di Wu and Ai-Dong Li*
National Laboratory of Solid State Microstructures and Department of Materials Science and Engineering, College of Engineering and Applied Sciences, Collaborative Innovation Center of Advanced Microstructures, Nanjing University, Nanjing 210093, People's Republic of China.
Correspondence and requests for materials should be addressed to Y.Q. C. (yqcao@nju.edu.cn) or A.D. L. (adli@nju.edu.cn)

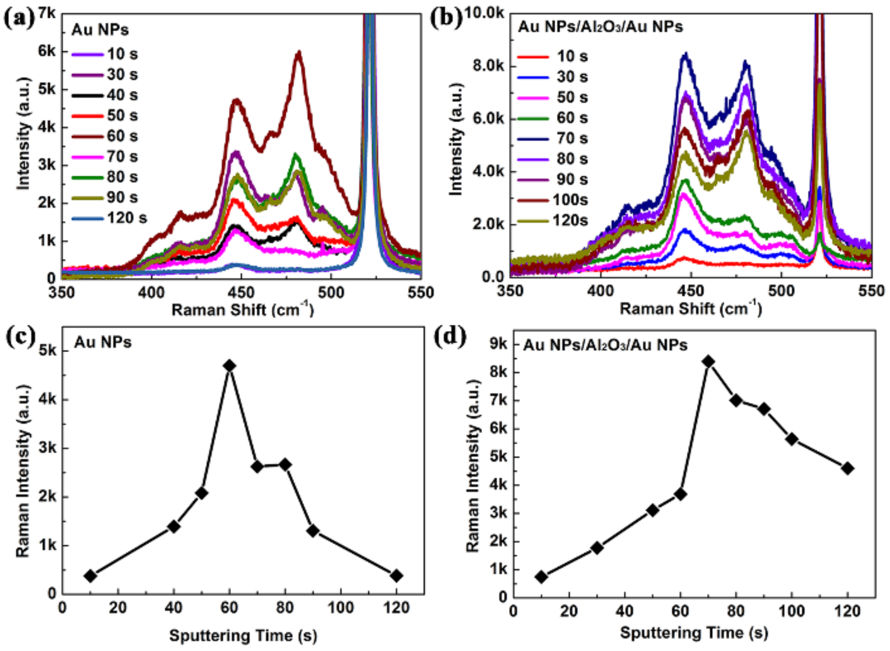

Figure S1. Raman spectra of MB molecules on (a) Au NPs and (b) Au NPs(60 s)/10 nm-Al_2O_3/Au NPs structure with various Au sputtering time. (c)(d) Corresponding dependence of Raman intensity at 446 cm^{-1} on the Au sputtering time.

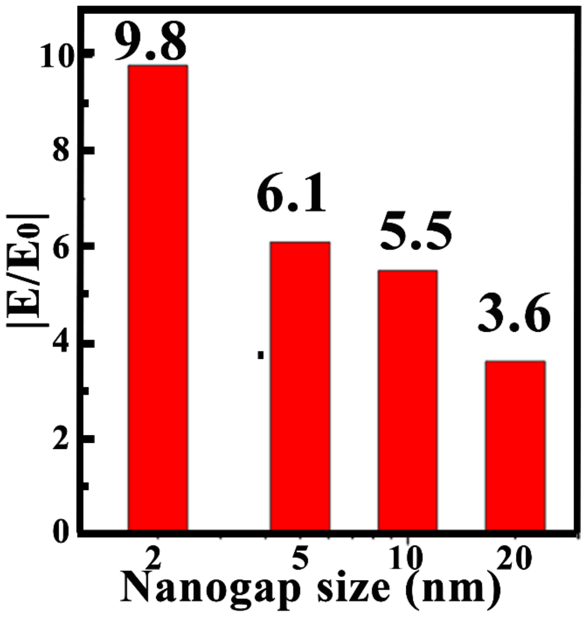

Figure S2. Comparison of FDTD simulation results of field enhancement factor $|E/E_0|$ at the gap area with various gap sizes from 2-20 nm.

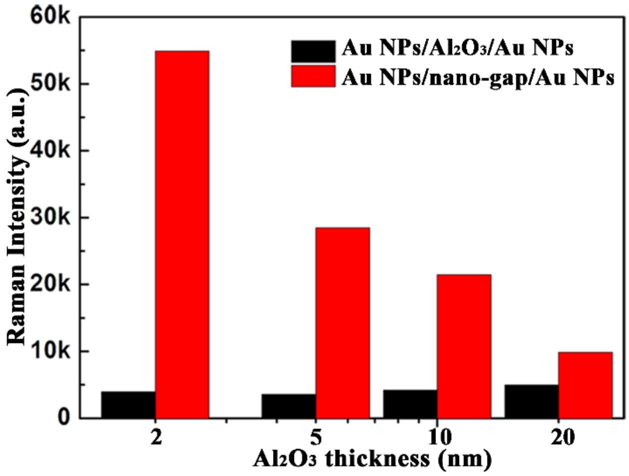

Figure S3. Comparison of Raman intensity (479 cm^{-1}) for Au NPs/Al_2O_3/Au NPs and Au NPs/nanogap/Au NPs.

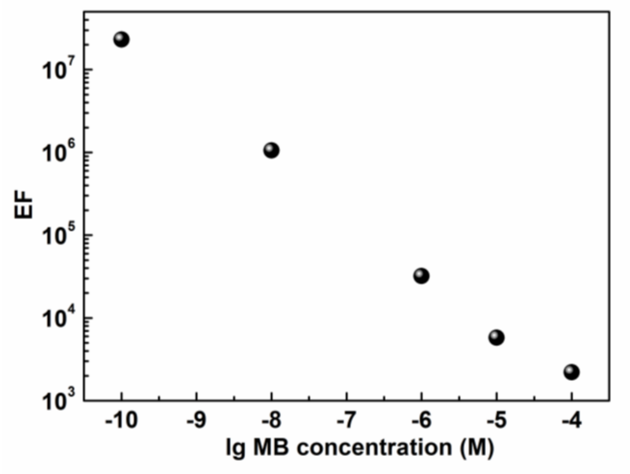

Figure S4. Enhancement factor of MB on Au NPs/nanogap (2 nm)/Au NPs for different MB concentration.

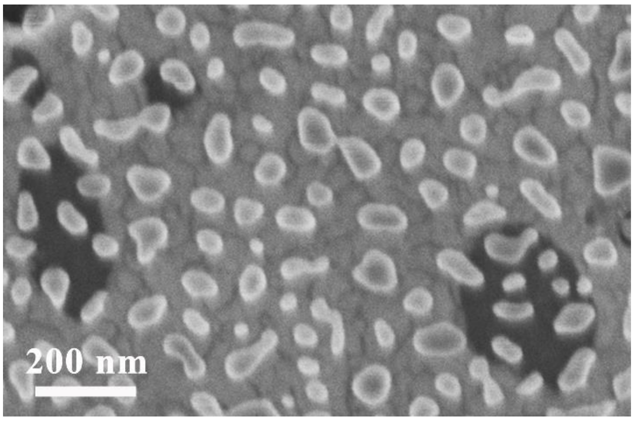

Figure S5. SEM image of Au NPs/nanogap/Au film.

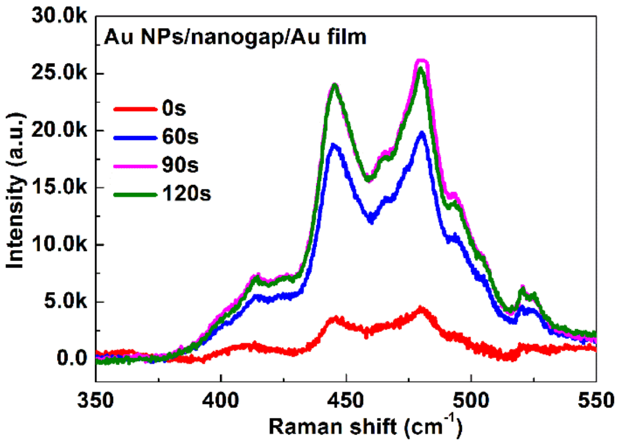

Figure S6. Raman spectra of MB molecules on Au NPs/Al_2O_3 (10 nm)/Au film with various etching time.
